# Editorial: The legacy of Sherrington and Adrian Nobel Prize: non-neuronal cells in information processing

**DOI:** 10.3389/fncel.2025.1634743

**Published:** 2025-06-10

**Authors:** Wuhyun Koh, Zhuofan Lei

**Affiliations:** ^1^Center for Cognition and Sociality, Institute for Basic Science (IBS), Daejeon, Republic of Korea; ^2^Department of Anesthesiology and Shock, Trauma and Anesthesiology Research Center (STAR), University of Maryland School of Medicine, Baltimore, MD, United States

**Keywords:** glial cells, astrocyte, gliotransmission, myelinating glial, glia-neuron interaction

In 1932, Sir Charles Scott Sherrington and Professor Edgar Douglas Adrian were jointly awarded the Nobel Prize in Physiology or Medicine for their foundational work on the physiology of neurons. Their discoveries, including the all-or-nothing principle of neural response and the mechanisms by which excitatory and inhibitory signals are integrated, laid the cornerstone of modern neuroscience. These insights shaped decades of thinking that positioned the neuron at the center of brain function, and they continue to guide contemporary approaches to understanding the nervous system.

Building on this legacy, neuroscience has steadily broadened its perspective. While neurons remain central to our understanding, an increasing body of evidence has revealed that non-neuronal cells, such as astrocytes, oligodendrocytes, and Schwann cells, also play essential and active roles in brain function (Verkhratsky and Butt, 2013). These glial cells, once regarded mainly as structural or metabolic support, are now recognized as regulators of synaptic signaling, contributors to circuit development, and modulators of behavior and plasticity. For example, thalamic astrocytes have been found to synthesize GABA, mediating tonic inhibition that supports synaptic integration, thus enabling tactile discrimination (Kwak et al., 2020). This finding highlights how astrocytes actively shape the fine-tuning of sensory information, underscoring their integral role in information processing within neural circuits. Extending the neuron-centric framework, this broader perspective enriches our understanding by incorporating the essential roles of non-neuronal cells, offering a more complete picture of how the brain processes information. Reflecting this expanded view, the Research Topic “*The Legacy of Sherrington and Adrian Nobel Prize: Non-Neuronal Cells in Information Processing*” honors and extends the groundbreaking work of Sherrington and Adrian. The four articles collected here cover a wide range of cellular mechanisms, offering compelling evidence that information processing in the brain emerges from the integrated activity of neuronal and non-neuronal elements.

Two studies focus on astrocytes (Lalo and Pankratov; Tavakoli et al.), which have become central to our understanding of glia-neuron interactions. Astrocytic Ca^2+^ dynamics, in particular, are known recognized as central to gliotransmission and synaptic plasticity (Semyanov et al., 2020). While inositol 1,4,5-trisphosphate receptor type 2 has been extensively studied as a major Ca^2+^ source from endoplasmic reticulum (ER) in astrocytes, Lalo et al. focus on the relatively underexplored role of ryanodine receptor (RyR). They demonstrate that RyR-mediated Ca^2+^-induced Ca^2+^ release from the ER substantially contributes to intracellular Ca^2+^ dynamics, particularly in astrocytic microdomains within the hippocampus and neocortex. This RyR-dependent signaling facilitates the release of gliotransmitters such as ATP and glutamate, which activate purinergic and glutamatergic currents in neighboring pyramidal neurons. In turn, this mechanism supports long-term synaptic potentiation, especially in aged animals. This astrocytic signaling not only modulates the strength of neuronal communication but also appears to enhance cognitive functions, suggesting a critical role for RyRs in glia-neuron interaction across the lifespan. Tavakoli et al. reveals that astrocyte Ca^2+^ activity in the dorsal striatum modulates cue-induced reinstatement of cocaine-seeking behavior, suggesting the role of astrocytes in addiction-related plasticity. Specifically, suppression of astrocytic Ca^2+^ via overexpression of a plasma membrane Ca^2+^ pump (PMCA, also known as CalEx) resulted in elevated neuronal Ca^2+^ transients and enhanced cocaine-seeking behavior after extinction. These findings indicate that astrocytic Ca^2+^ acts as a brake on striatal excitability and relapse-like behavior, especially following drug experience. Together, their findings underscore the increasingly recognized role of astrocytes as active modulators of neural circuit function, capable of shaping synaptic signaling and behavioral outcomes through finely tuned Ca^2+^ dynamics.

Two other studies focus on myelinating glial cells (Butler and Dale; Hawker et al.). Butler and Dale investigate Connexin32 hemichannels in Schwann cells, particularly in the context of X-linked Charcot-Marie-Tooth disease. Several mutations in Connexin32, which is prominently expressed in myelinating Schwann cells, are shown to disrupt CO_2_ sensitivity and impair ATP release, suggesting a loss of critical signaling capacity that may contribute to the disease. These findings reinforce the idea that ion channels in glial cells, particularly Schwann cells, play essential roles in environmental sensing and maintaining neural function. In parallel, Hawker et al. establish *ex vivo* spinal cord model of demyelination and remyelination, providing a platform for studying how oligodendrocyte precursor cells repair damaged myelin. This model replicates key aspects of injury and regeneration and holds promise for developing treatments for conditions such as multiple sclerosis and spinal cord injury.

Taken together, these studies support a conceptual shift in neuroscience. Rather than viewing neurons as isolated information processors, neural circuits are increasingly recognized as dynamic networks shaped by interactions among diverse cell types. Glial cells contribute to the timing, precision, and adaptability of synaptic signaling through Ca^2+^ dynamics, gliotransmitter release, and metabolic coupling. This broader framework builds on the foundational insights of Sherrington and Adrian, extending our understanding from single-neuron activity to the integrated cellular interactions that underlie cognition, behavior, and brain health.

This Research Topic offers a reflection on how far the field has come, and where it may be headed. By exploring the roles of non-neuronal cells in information processing and cognition, we deepen our understanding of brain complexity and move closer to integrative solutions for neurological disorders. Sherrington and Adrian helped us understand how neurons communicate each other. Today, we are uncovering how non-neuronal cells in the brain contribute to that ongoing conversation.
